# Supplementary material for: Phosphoserine for the generation of lanthanide-binding sites on proteins for paramagnetic nuclear magnetic resonance spectroscopy
Source: Magn Reson (Gott). 2021 Jan 6;2(1):1–13. doi: 10.5194/mr-2-1-2021 (PMC10539748; doi:10.5194/mr-2-1-2021)
Supplement: The supplement related to this article is available online at: https://doi.org/10.5194/mr-2-1-2021-supplement. [file mr-2-1-supplement.pdf]

Supplement of Magn. Reson., 2, 1–13, 2021
https://doi.org/10.5194/mr-2-1-2021-supplement
© Author(s) 2021. This work is distributed under
the Creative Commons Attribution 4.0 License.

Supplement of

Phosphoserine for the generation of lanthanide-binding sites on proteins for paramagnetic nuclear magnetic resonance spectroscopy

Sreelakshmi Mekkattu Tharayil et al.

Correspondence to: Gottfried Otting (gottfried.otting@anu.edu.au)

The copyright of individual parts of the supplement might differ from the CC BY 4.0 License.

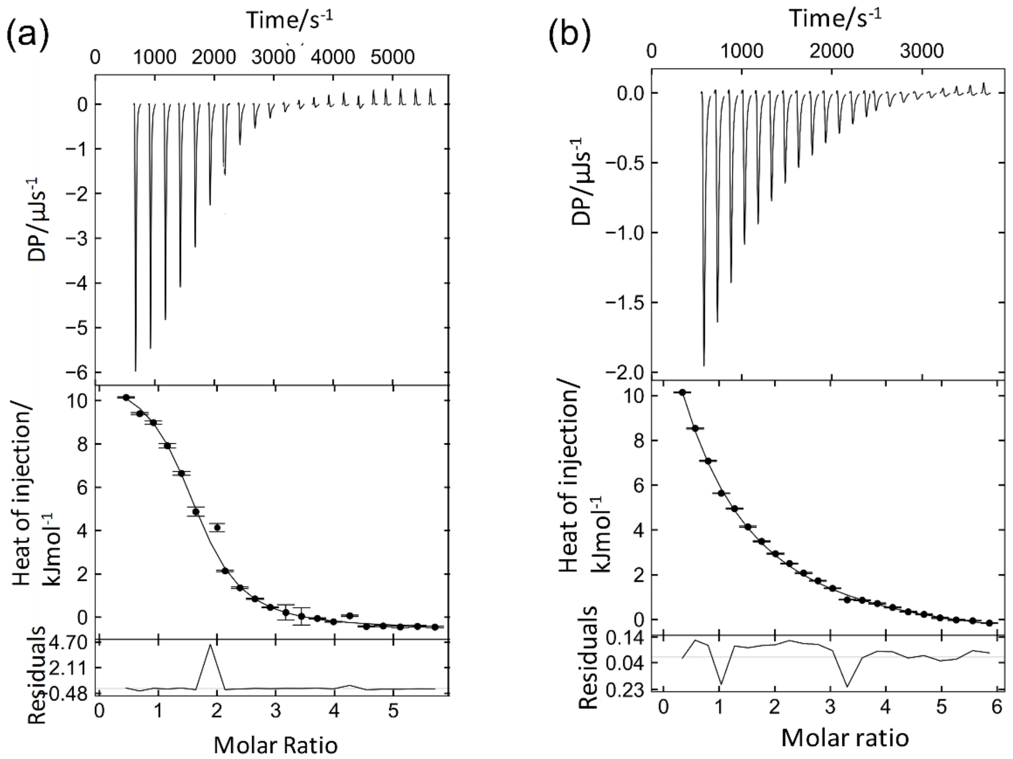

Figure S1. Representative isothermal titration calorimetry experiments of ubiquitin E18Sep titrated with $LnCl_3$. (a) Cell = 150 μM ubiquitin E18Sep; syringe = 2.7 mM $TbCl_3$. (b) Cell = 150 μM ubiquitin E18Sep; syringe = 2.7 mM $TmCl_3$. The top panel shows the baseline-corrected power traces. The middle panel displays the heat data and best fit. The bottom panel shows the residual of the fit. Error bars calculated by the program NITPIC (Keller et al., 2015) indicate the standard error in the integration of the peaks. DP denotes the power differential between the reference and sample cells to maintain a zero temperature difference between the cells.

Values for the dissociation constant K_d were derived from global fits to data from two and three different measurements with Tb^{3+} and Tm^{3+}, respectively. Fits were performed either with inclusion of the binding stoichiometry n as a fitting parameter or setting $n = 1$, with the result shown underneath.

Fitted parameters	Tb^{3+}		Tm^{3+}	
	setting $n = 1$	fitting n^a	setting $n = 1$	fitting n^b
ΔH (kJ mol^{-1})	15	23	20	12
ΔS ($Jmol^{-1}K$)	137	161	143	128
K_d (μM)	25	42	133	32

[a] The fit yielded $n = 0.7$.
[b] The fit yielded $n = 1.4$.

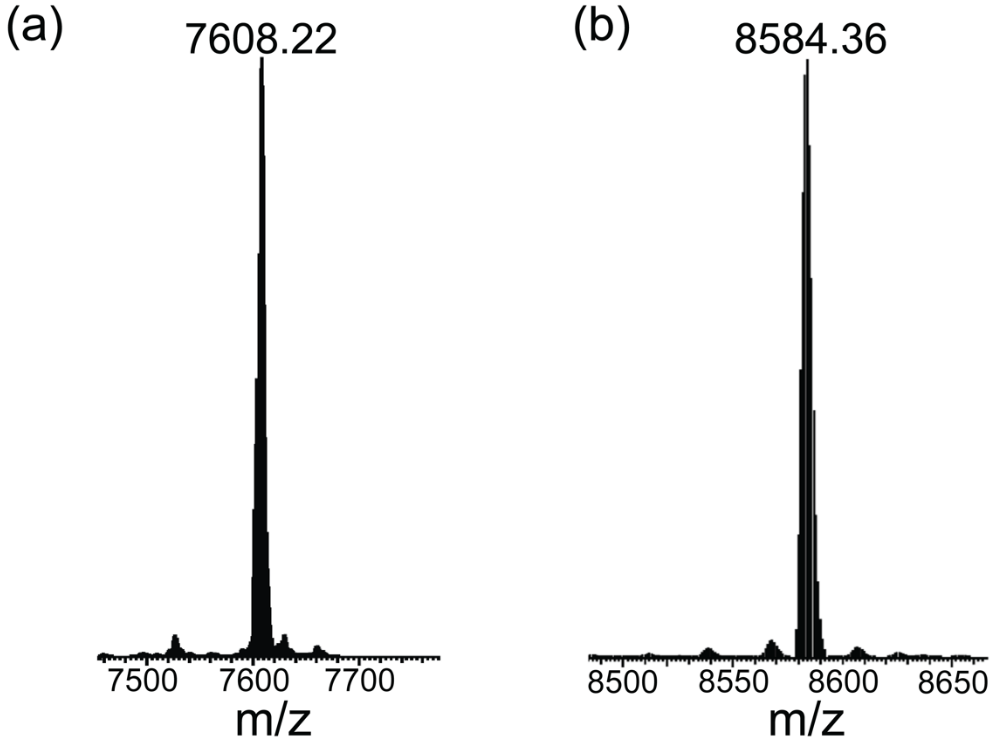

Figure S2. Mass spectra of intact GB1 protein with two Sep residues, confirming the double amber suppression. (a) GB1 K10Sep/T11Sep after cleavage of the His_6-tag with TEV protease. The expected mass is 7610 Da. (a) GB1A24Sep/K28Sep before cleavage of the His_6-tag. High purity of the protein was achieved already by a single affinity chromatography step using Ni-NTA. The expected mass is 8584.69 Da.

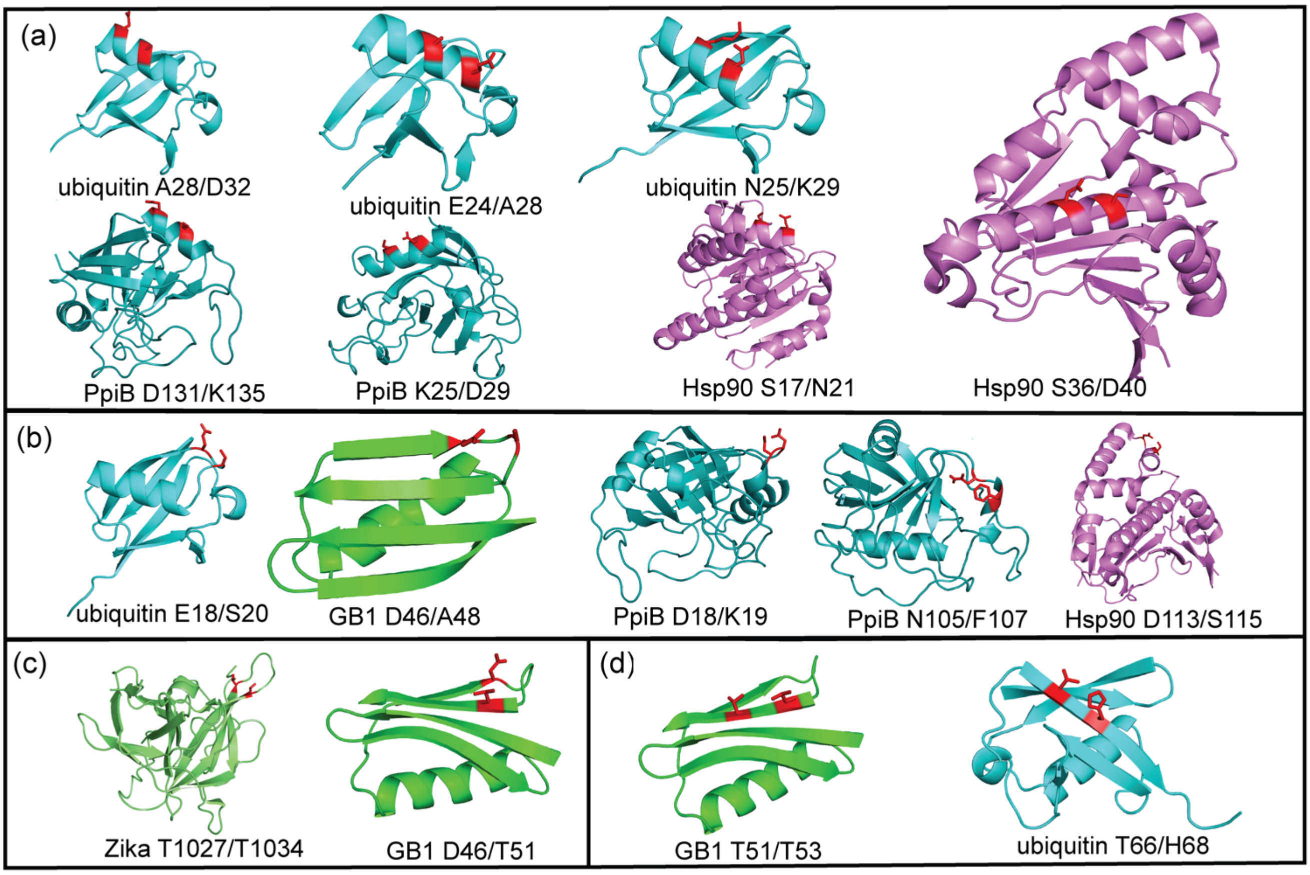

Figure S3. Double-amber mutants tested for expression with phosphoserine. Mutation sites are highlighted in red, showing the side chains of the wild-type protein in stick representation. Ubiquitin is abbreviated Ubi. The Zika virus NS2B-NS3 protease is denoted Zika. (a) Targeted sites in positions *i* and *i*+4 of an α-helix. (b) Targeted sites in positions *i* and *i*+2 of a loop region. (c) Targeted sites located in two neighbouring β-strands. (d) Targeted sites in positions *i* and *i*+2 of a β-strand.

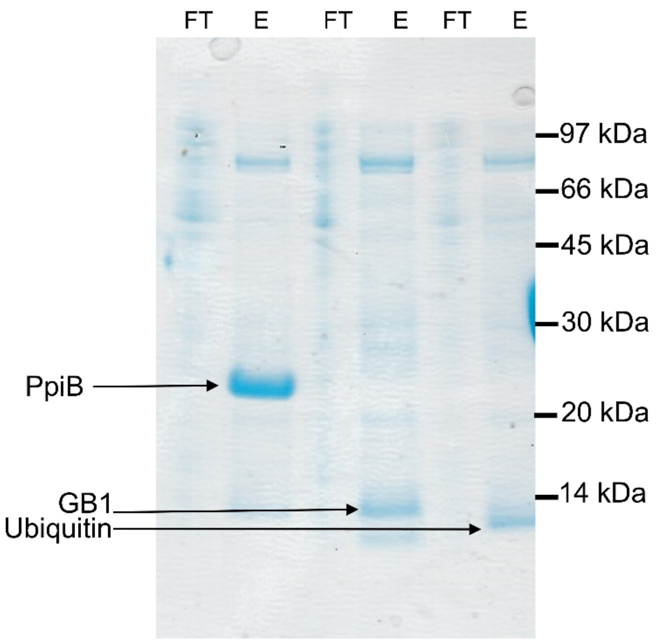

Figure S4. SDS-PAGE gel illustrating the successful production and Ni-NTA column purification of PpiB K25BoK/D29BoK, GB1 T51BoK/T53BoK and ubiquitin A28BoK/D32BoK, where BoK stands for Boc-lysine. The lanes labelled FT and E are of the flow-through and elution fractions, respectively. Arrows identify the bands of the full-length proteins.

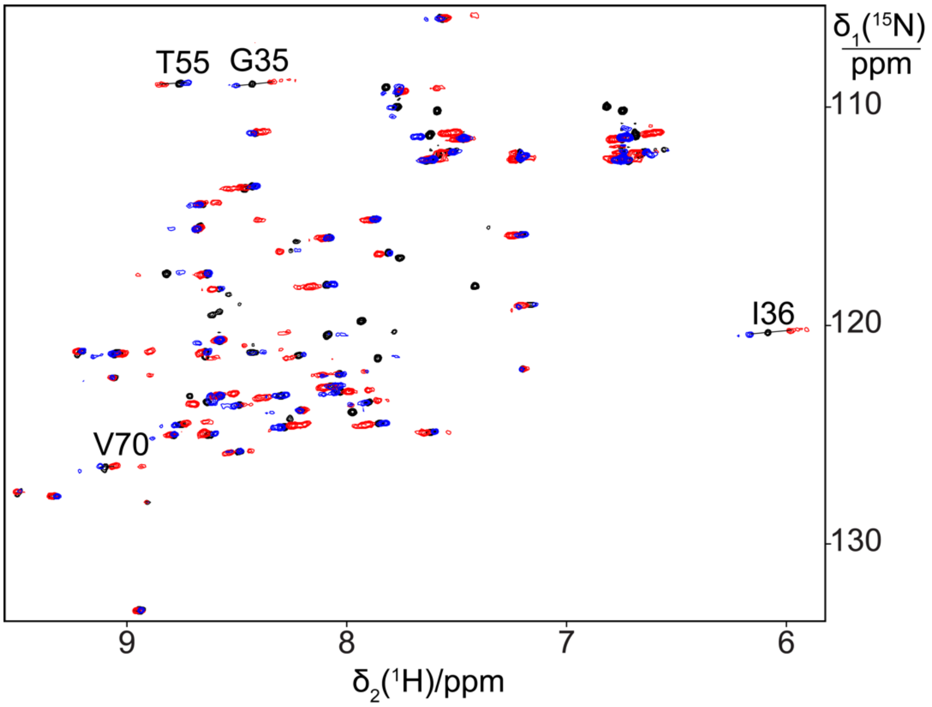

Figure S5. Small PCSs generated by lanthanides in the ubiquitin Q2D/E64Sep mutant indicate the absence of a well-defined lanthanide binding site. The figure shows a superimposition of [^{15}N,^{1}H]-HSQC spectra of 0.3 mM solutions of ubiquitin Q2D/E64Sep recorded in the presence of Tb^{3+} (red), Tm^{3+} (blue) or Y^{3+} (black).

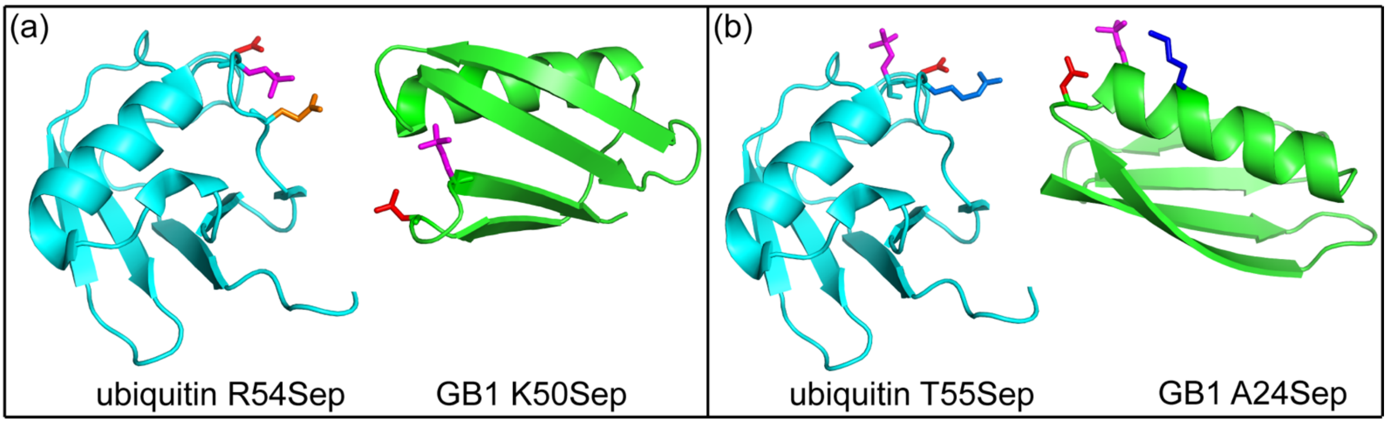

Figure S6. Single-phosphoserine mutants of ubiquitin and GB1, which either did not express or expressed but did not produce PCSs upon titration with lanthanide ions. The side chains of selected residues are highlighted, with phosphoserine in magenta, aspartate in red, glutamate in orange and lysine residues in blue. (a) Mutants that failed to express. In the ubiquitin mutant R54Sep, D58 and E51 are near the Sep residue in position 54. In the GB1 mutant K50Sep, D7 is near the Sep residue in position 50. (b) Mutants that did not produce PCSs upon titration with lanthanide ions. In the ubiquitin mutant T55Sep, R54 can form a salt bridge with D58 or Sep55. In the GB1 mutant A24Sep, K28 can form a salt bridge with the Sep residue in position 24, while D22 does not have a salt bridge partner.

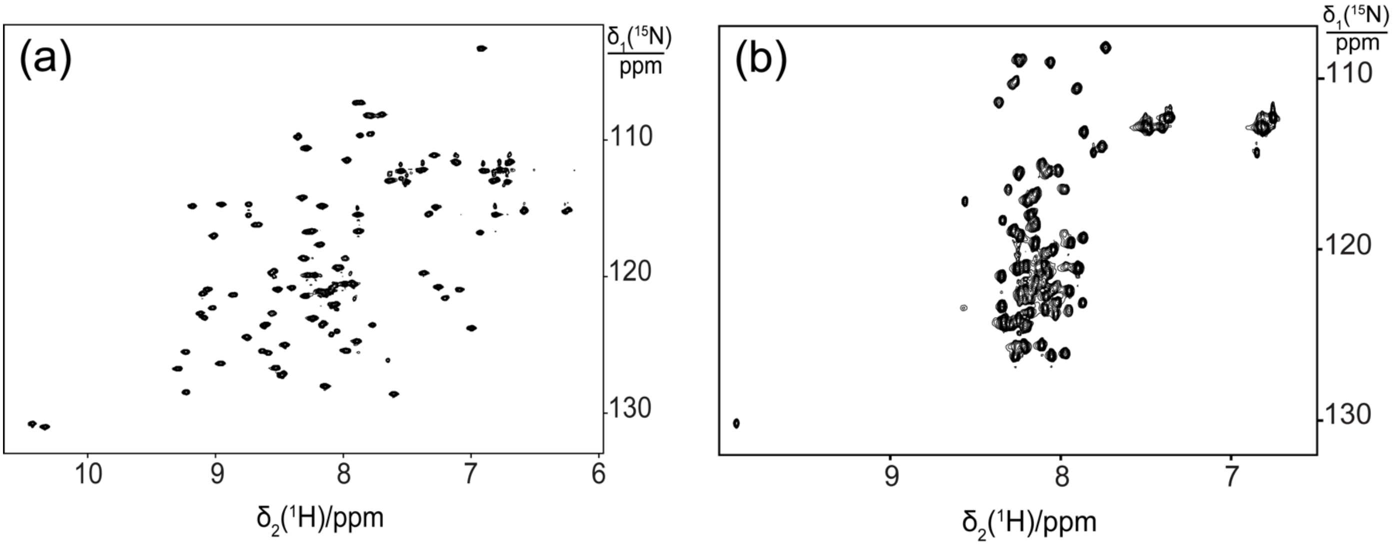

Figure S7. The introduction of negatively charged residues in close proximity can lead to protein unfolding. The figure shows [^{15}N,^{1}H]-HSQC spectra of 0.3 mM solutions of (a) wild-type GB1 and (b) GB1 K4D/I6Sep in 20 mM HEPES-KOH, pH 7.0. The spectrum was recorded at a ^{1}H-NMR frequency of 800 MHz.

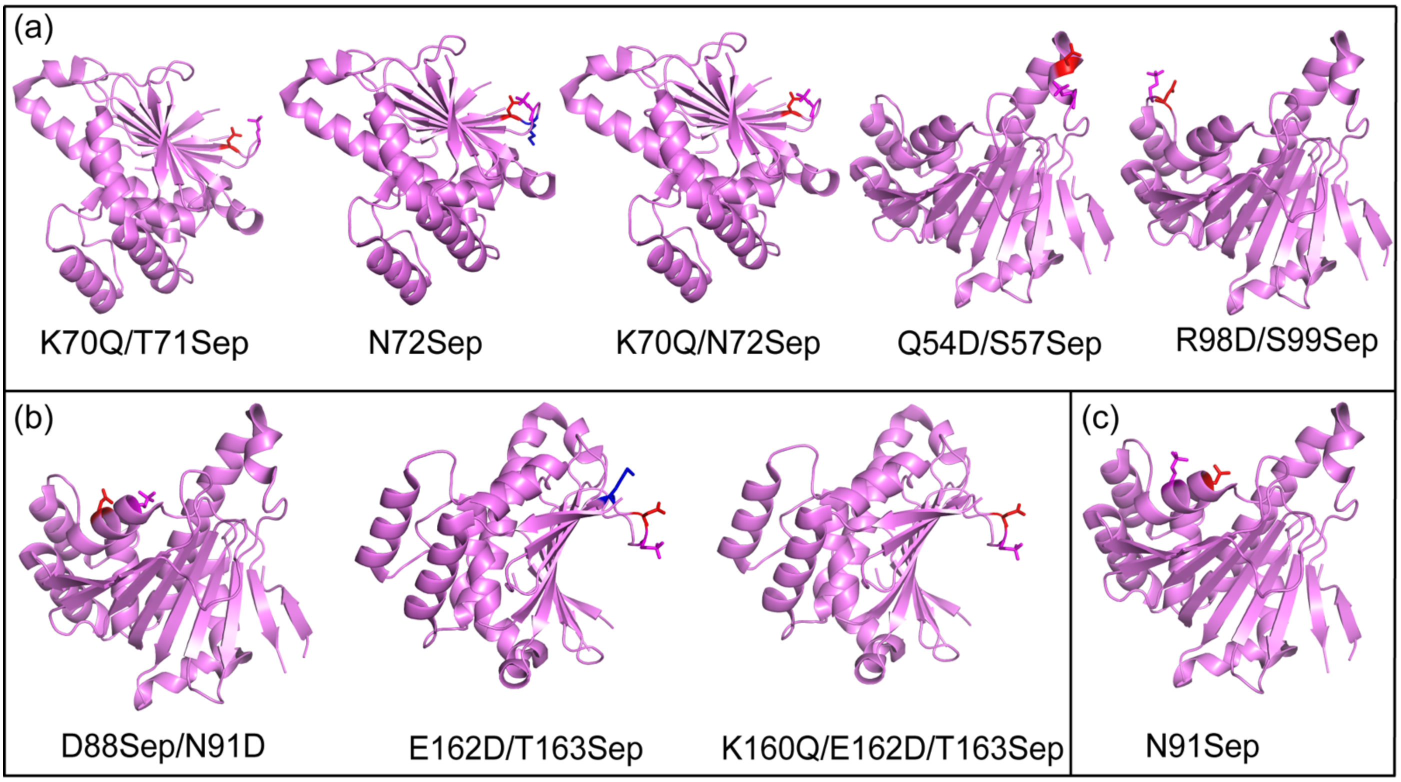

Figure S8. Single-phosphoserine mutants of *Pf* Hsp90-N tested for expression with phosphoserine. The side chains of selected residues are highlighted as in Figure S7. (a) Mutants that failed to express. (b) Mutants that were produced only in yields too low for NMR spectroscopy. (c) Mutant that expressed in sufficient yield for isotope labelling. The Sep residue in position 91 was expected to form a lanthanide binding site together with D88 (highlighted in red). The residues targeted for mutation sites were chosen by their sidechains pointing in the same direction and the absence of positively charged residues nearby with the potential for making a salt-bridge with either the aspartate or phosphoserine residue in the mutant protein.

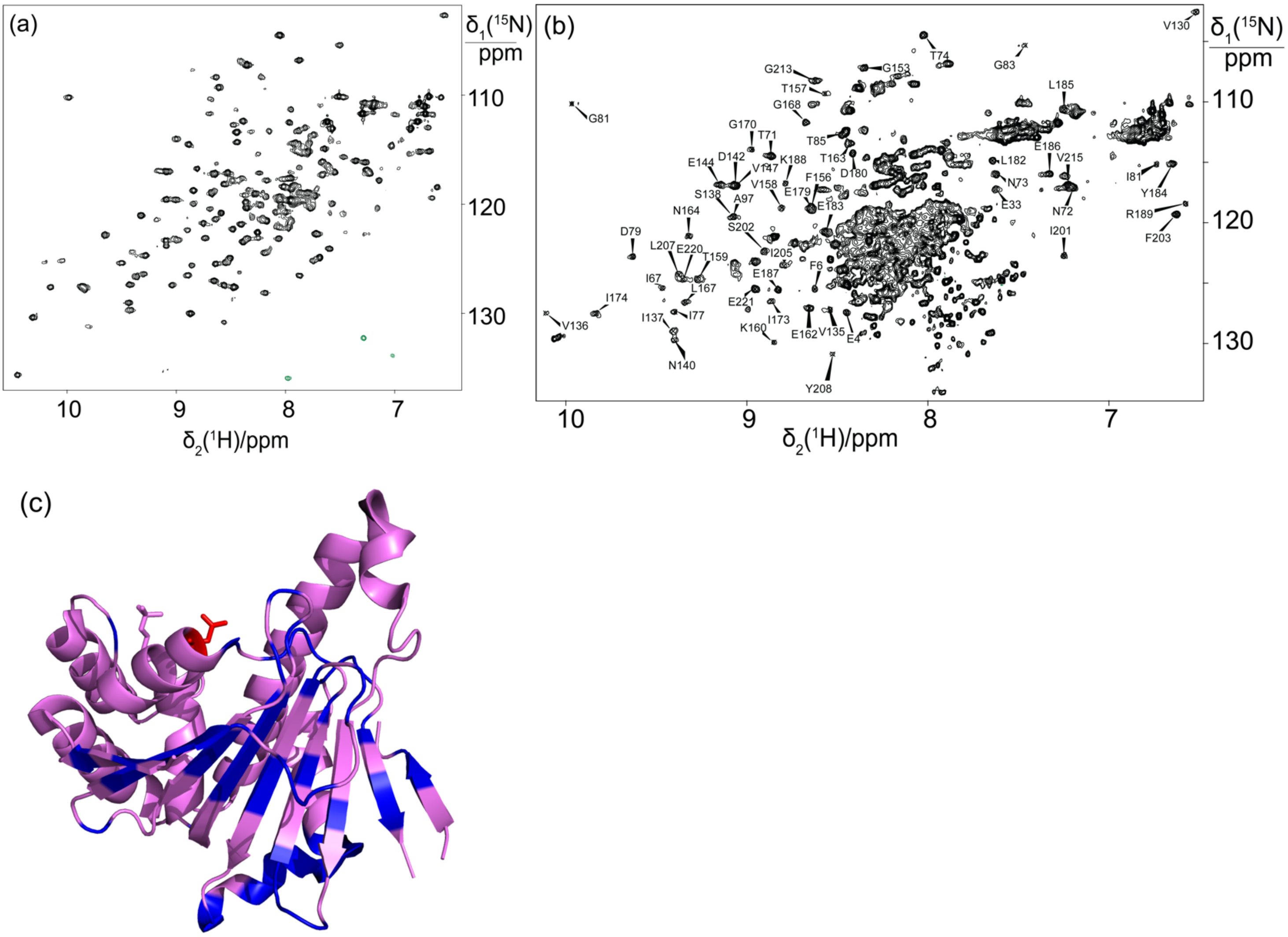

Figure S9. Partial unfolding of the *Pf*Hsp90-N N91Sep mutant evidenced by NMR spectroscopy. (a) [^{15}N,^{1}H]-HSQC spectrum of a 290 µM solution of ^{15}N-labelled wild-type *Pf*Hsp90-N in 20 mM MES-KOH, pH 6.5, 100 mM NaCl and 1 mM DTT. The spectrum was recorded at a ^{1}H NMR frequency of 600 MHz. (b) Same as (a), but of a 280 µM solution of the mutant N91Sep. Cross-peaks conserved between the spectra of the wild-type and mutant proteins are identified. (c) Ribbon representation of the crystal structure of *Pf*Hsp90-N (PDB ID 3K60; Corbett and Berger, 2010). Highlighted in blue are the amino-acid residues with conserved chemical shifts in the mutant and wild-type samples. The side chains of D88 and the Sep residue in position 91 are coloured red and magenta, respectively.

Table S1 PCSs of backbone amide protons measured with $TbCl_3$ and $TmCl_3$ in different ubiquitin mutants[a]

ubiquitin E18Sep				ubiquitin E16Q/E18Sep				ubiquitin T22Sep/N25D/K29Q	
Tb^{3+}		Tm^{3+}		Tb^{3+}		Tm^{3+}		Tb^{3+}	
Residue[b]	PCS /ppm	Residue[b]	PCS /ppm	Residue[b]	PCS /ppm	Residue[b]	PCS /ppm	Residue[b]	PCS /ppm
Lys6	-0.452	Val5	0.160	Phe4	-0.326	Phe4	0.229	Phe4	-0.116
Thr9	-0.253	Lys6	0.099	Lys6	-0.424	Val5	0.318	Lys6	-0.153
Lys11	-0.287	Thr7	0.077	Thr7	-0.363	Lys6	0.193	Thr7	-0.125
Thr12	-0.363	Leu8	0.058	Thr9	-0.241	Thr7	0.149	Leu8	-0.076
Ile13	-0.625	Thr9	0.051	Lys11	-0.277	Leu8	0.110	Thr9	-0.049
Glu34	-0.623	Lys11	0.058	Thr12	-0.349	Thr9	0.101	Lys11	-0.086
Gly35	-0.437	Thr12	0.073	Ile13	-0.567	Lys11	0.119	Thr12	-0.126
Ile36	-0.380	Ile13	0.125	Gly35	-0.218	Thr12	0.145	Ile13	-0.224
Gln40	-0.235	Gly35	0.072	Ile36	-0.361	Ile13	0.247	Glu34	-0.292
Gln41	-0.389	Ile36	0.068	Gln40	-0.186	Glu34	0.260	Gly35	-0.173
Ile44	-0.399	Gln41	0.059	Gln41	-0.334	Gly35	0.173	Ile36	-0.095
Phe45	-0.247	Leu43	0.088	Ile44	-0.379	Ile36	0.155	Gln41	0.144
Ala46	-0.071	Ile44	0.084	Ala46	-0.080	Gln40	0.085	Leu43	-0.031
Gly47	-0.069	Phe45	0.053	Gly47	-0.084	Gln41	0.127	Ile44	-0.076
Lys48	-0.136	Ala46	0.033	Lys48	-0.142	Ile44	0.171	Phe45	-0.124
Asn60	0.633	Gly47	0.029	Leu50	-0.384	Ala46	0.065	Ala46	-0.083
Gln62	0.974	Lys 48	0.032	Tyr59	-0.044	Gly47	0.064	Leu50	-0.064
Glu64	0.631	Leu50	0.071	Asn60	0.422	Lys48	0.067	Tyr59	-0.213
His68	-0.358	Asp52	0.063	Ile61	0.760	Leu50	0.138	Ile61	0.145
Gly75	-0.050	Arg54	0.131	Gln62	0.870	Tyr59	-0.013	Gln62	0.190
		Thr55	0.106	Glu64	0.726	Asn60	-0.122	Glu64	0.197
		Tyr59	-0.034	Leu67	-0.42	Ile61	-0.147	Ser65	0.096
		Asn60	-0.091	His68	-0.337	Gln62	-0.141	Thr66	-0.013
		Ile61	-0.094	Val70	-0.335	Glu64	-0.078	Leu67	-0.170
		Gln62	-0.082	Arg74	-0.063	Ser65	-0.013	His68	-0.138
		His68	0.083	Phe4	-0.326	Leu67	0.217	Gly76	0.054
		Gly75	0.013	Lys6	-0.424	His68	0.165		
						Val70	0.142		

[a] Data recorded at 25 °C and pH 7.0.

Table S2 PCSs of backbone amide protons generated with $TbCl_3$ and $TmCl_3$ in different GB1 mutants[a]

GB1K10D/T11Sep		GB1A24Sep/K28Sep				GB1K10Sep/T11Sep	
Tb^{3+}		Tb^{3+}		Tm^{3+}		Tb^{3+}	
Residue[b]	Residue[b]	Residue[b]	PCS[exp]/ppm	Residue[b]	PCS[exp]/ppm	Residue[b]	PCS[exp]/ppm
Thr2	-0.043	Tyr3	-2.769	Tyr 3	1.181	Met1	-0.174
Lys4	-0.143	Lys4	-2.685	Lys4	1.223	Thr2	-0.235
Leu5	-0.230	Ile6	-1.964	Ile6	0.972	Tyr 3	-0.353
Ile6	-0.507	Leu7	-0.939	Leu7	0.466	Lys4	-0.554
Glu15	-0.219	Asn8	-0.88	Gly9	0.232	Leu5	-0.948
Thr17	-0.069	Gly9	-0.398	Lys10	0.127	Thr16	-1.200
Thr18	-0.057	Lys10	-0.158	Thr11	0.039	Thr17	-0.597
Glu19	-0.007	Thr11	-0.011	Leu12	0.056	Thr18	-0.439
Ala20	-0.015	Leu12	-0.053	Lys13	0.101	Glu19	-0.240
Asp22	-0.014	Lys13	-0.172	Gly14	0.266	Ala20	-0.205
Ala26	-0.020	Gly14	-0.504	Gly15	0.260	Val21	-0.125
Glu27	-0.030	Glu15	-0.538	Thr16	0.563	Asp22	-0.141
Val29	0.097	Thr16	-1.187	Thr17	0.488	Ala23	-0.151
Gln32	0.387	Thr17	-1.144	Tyr33	-1.230	Ala24	-0.118
Tyr45	-0.554	Glu19	-2.346	Ala34	-0.425	Ala26	-0.193
Asp46	-0.299	Ala20	-3.352	Asn35	-1.241	Glu27	-0.218
Asp47	-0.162	Tyr33	2.919	Asp36	-1.329	Lys28	-0.125
Thr49	-0.147	Ala34	1.304	Asn37	-0.647	Val29	-0.185
Lys50	-0.140	Asn35	3.086	Gly38	-0.580	Phe30	-0.356
Phe52	-0.302	Asp36	3.103	Gly41	0.483	Tyr32	-0.097
		Asn37	1.587	Glu42	0.682	Val39	0.204
		Gly38	1.444	Trp43	1.561	Asp40	1.044
		Val39	0.870	Tyr45	1.210	Tyr45	-0.380
		Gly41	-0.748	Asp46	1.012	Asp46	-0.484
		Glu42	-1.292	Ala48	0.204	Asp47	-0.266
		Trp43	-2.998	Thr49	0.392	Ala48	-0.266
		Tyr45	-2.103	Lys50	0.547	Thr49	-0.339
		Asp46	-2.073	Phe52	1.526	Lys50	-0.340
		Ala48	-0.280	Val54	0.894	Thr51	-0.477
		Thr49	-0.839	Thr55	0.776	Phe52	-0.808
		Lys50	-1.182	Glu56	0.357	Thr53	-1.037
		Val54	-1.745				
		Thr55	-1.457				
		Glu56	-0.646				

[a] Data recorded at 25 °C and pH 7.0.

References

Corbett, K. D. and Berger, J. M.: Structure of the ATP-binding domain of *Plasmodium falciparum* Hsp90, Proteins, 78, 2738–2744, https://doi.org/10.1002/prot.22799, 2010.

Keller, S., Vargas, C., Zhao, H., Piszczek, G., Brautigam, C. A., and Schuck, P.: High-precision isothermal titration calorimetry with automated peak-shape analysis, Anal. Chem., 84, 5066–5073, https://doi.org/10.1021/ac3007522, 2012.
